# Lack of a putative intrinsically disordered protein anchored to the sporangiospore cell wall causes fragile sporangium formation in Actinoplanes missouriensis

**DOI:** 10.1099/mic.0.001656

**Published:** 2026-01-14

**Authors:** Zhuwen Tan, Takeaki Tezuka, Yasuo Ohnishi

**Affiliations:** 1 Department of Biotechnology, Graduate School of Agricultural and Life Sciences, The University of Tokyo, Bunkyo-ku, Tokyo, Japan; 2 Collaborative Research Institute for Innovative Microbiology, The University of Tokyo, Bunkyo-ku, Tokyo, Japan

**Keywords:** *Actinoplanes missouriensis*, cell surface protein, sortase, sporangiospore, sporangium formation, sporangium matrix

## Abstract

The filamentous actinomycete *Actinoplanes missouriensis* develops terminal sporangia on substrate mycelia via short sporangiophores. Each sporangium, surrounded by an outer envelope, contains a few hundred spores encapsulated by the sporangium matrix. In this study, we identified a spore surface-displayed protein, SspA, that is required for the structural strength of sporangia in *A. missouriensis* through suppressor screening using a spore release-deficient mutant. SspA has a sortase-dependent cell wall-localizing signal, and its mature part is predicted to be a putative intrinsically disordered protein. An *sspA* null mutant (Δ*sspA*) strain formed sporangia, but the mutant sporangia were highly fragile and collapsed immediately to release spores when suspended in aquatic solutions. Transmission electron microscopy revealed that the Δ*sspA* sporangia did not mature normally; the electron-dense sporangium matrix was not observed in the peripheral region of each spore, and the outer envelope of some sporangia was damaged. Peptide-tagged SspA proteins produced in the Δ*sspA* strain were detected on the surface of the zoospores using the HiBiT system. The heat tolerance of Δ*sspA* zoospores was higher than that of wild-type zoospores, suggesting that SspA influences the frequency of cross-bridges in the cell wall peptidoglycan. Phenotypic changes in the Δ*sspA* strain were restored by introducing *sspA* with its own promoter into the Δ*sspA* strain. These results demonstrate that SspA is a sporangiospore cell wall-anchored protein required for the formation of rigid sporangium structures in *A. missouriensis*. It is speculated that SspA is involved in the production of the sporangium matrix polysaccharides.

## Data Availability

Nucleotide sequence data of genome sequencing analysis were deposited in the DDBJ Sequence Read Archive under the accession numbers SAMD01693174 (Δ*asfR* strain), SAMD01693176 (S-1 strain) and SAMD01693175 (S-2 strain). The project accession number of this analysis is PRJDB37713.

## Introduction

In gram-positive bacteria, the cell wall acts as a scaffold for a wide variety of surface-displayed proteins, which play pivotal roles in cell physiology by enabling each bacterium to effectively interact with its surrounding environment. Many of these surface proteins are anchored to the cell wall by a group of membrane-bound cysteine transpeptidases called sortases, which catalyse the covalent bond formation between the surface proteins and cell wall peptidoglycan [1,3]. The substrate proteins of sortases contain a sorting signal consisting of a C-terminal pentapeptide recognition motif (the LPXTG motif) followed by a transmembrane helix and a stretch of positively charged amino acids [4]. Sortases catalyse the cleavage of substrate proteins within the pentapeptide motif (between fourth T and fifth G) to form a stable thioacyl intermediate, which is relieved by the nucleophilic attack of an amino group in a peptidoglycan precursor, lipid II. Thus, proteins covalently attached to the peptidoglycan precursor are incorporated into the growing peptidoglycan at the cell surface via a transglycosylation reaction [5,8].

Actinomycetes are gram-positive, mainly soil-dwelling bacteria that typically exhibit filamentous growth and complex morphological development. Morphological development in the representative genus *Streptomyces* has been well studied. The model species *Streptomyces coelicolor* A3(2) forms substrate mycelia during vegetative growth and then produces aerial hyphae that emerge from the substrate mycelia, culminating in spore chains [9,10]. In *S. coelicolor* A3(2), a group of surface proteins called chaplins plays a crucial role in enabling aerial hyphae to grow into the air [11,12]. Among the eight proteins of the chaplin family, three long chaplins (ChpA–C) include a C-terminal sorting signal and localize to the cell wall to promote the assembly of five short chaplins (ChpD–H) on the emerging aerial surfaces [13,14]. Consistent with this, the formation of aerial hyphae was severely delayed in a double mutant of two sortase genes, *srtE1* and *srtE2* [14]. To the best of our knowledge, ChpA–C are the only sortase-dependent surface proteins that have been functionally characterized in *Streptomyces*.

*Actinoplanes missouriensis* is a filamentous actinomycete with a complex morphology. It forms branched substrate mycelia during vegetative growth and produces globose or subglobose terminal sporangia on short sporangiophores from substrate mycelia when cultivated on a nutrient-poor agar medium, such as humic acid-trace element (HAT) agar. Each sporangium contains a few hundred spherical flagellated spores, and the space between the spores inside a sporangium is filled with an intrasporangial matrix (sporangium matrix). The outer envelope of the sporangium is composed of a three-layer membranous structure [15]. Upon contact with water, sporangia open to release spores through a process known as sporangium dehiscence [16]. As the spores swim in an aquatic environment using flagella, they are termed zoospores after being released from sporangia [17,18]. Zoospores stop swimming and germinate in a niche suitable for vegetative growth [19]. When cultivated on HAT agar at 30 °C, small sporangium-like structures are generated after 2–3 days of cultivation. Mature sporangia that can release spores under sporangium dehiscence-inducing conditions are formed after 5–7 days of cultivation.

Recently, we reported that an orphan response regulator receiver domain protein of the two-component regulatory system, named AsfR, is involved in the formation of mature sporangia in *A. missouriensis* [20]. An *asfR* null mutant (Δ*asfR*) strain produces morphologically normal sporangia, as well as somewhat irregularly shaped sporangia. However, spores were scarcely released from the Δ*asfR* sporangia, indicating that *asfR* is involved in the formation of physiologically matured sporangia that can release spores under sporangium dehiscence-inducing conditions. Although we showed that an amino acid replacement at the predicted phosphorylation site (D72N) rendered AsfR non-functional, the molecular functions of AsfR, as well as proteins that function with AsfR, remain to be elucidated [20]. In this study, to obtain insights into the physiological roles of AsfR, we isolated suppressor strains from the Δ*asfR* strain, whose sporangia released much larger numbers of spores than those from the Δ*asfR* sporangia, and analysed the mutations generated in the suppressor strains. Through a genetic study, we identified *AMIS_68180*, which encodes a sortase-dependent cell wall-anchored protein, as a gene responsible for the release of spores from sporangia in one of the suppressor strains. We designated AMIS_68180 as SspA (spore surface protein A) based on its localization revealed in this study. We functionally characterized SspA and demonstrated that it is required for the structural strength of sporangia in * A. missouriensis*. SspA does not seem to be related, at least directly, to the function of AsfR. We also discuss the putative function of SspA, which is the first structural protein, not transcriptional regulators and enzymes, reported to be involved in morphological development in *A. missouriensis*.

## Methods

### General methods

The bacterial strains, plasmid vectors and media used in this study were described previously [21,24]. The primers used in this study are listed in Table S1 (available in the online Supplementary Material). *A. missouriensis* and *Escherichia coli* were cultivated as previously described [25]. Scanning electron microscopy (SEM) was performed using an S-4800 electron microscope (Hitachi, Tokyo, Japan), as described previously [26]. Transmission electron microscopy (TEM) was performed using an H-7600 electron microscope (Hitachi), as described previously [27]. Phase-contrast microscopy of sporangia and mycelia was performed using a BH-2 phase-contrast microscope (Olympus, Tokyo, Japan), as described previously [28]. Zoospores released from sporangia were quantified as described previously [29].

### Isolation of suppressor strains

First, we generated a mutant library of the Δ*asfR* strain as follows. The Δ*asfR* strain was inoculated and cultivated on HAT agar at 30 °C for 7 days for sporangium formation. Sporangium dehiscence is usually induced by suspending sporangia harvested from the HAT agar surface in 25 mM histidine solution, followed by incubation for 1 h. However, the number of spores released from Δ*asfR* sporangia was quite low, as reported previously [20]. Therefore, sporangia and mycelia were harvested from the agar surface and suspended in 25 mM histidine solution, followed by mixing with glass beads using a vortex mixer to forcibly induce spore release. After mixing at room temperature for 1 h, the solution was filtered through a 5-µm membrane filter (Pall Corporation, NY, USA) to eliminate mycelia and sporangia. The resulting spore suspension was irradiated with UV light until the survival rate reached ~30% to generate a zoospore mutant library. Next, mutant strains, whose sporangia opened to release spores in 25 mM histidine solution, were enriched as follows: (i) the irradiated spore suspension was inoculated onto HAT agar and cultivated at 30 °C for 7 days to induce sporangium formation; (ii) sporangia were harvested from the agar surface and suspended in 25 mM histidine solution, followed by rotation at room temperature for 1 h to induce sporangium dehiscence; and (iii) the suspension was filtered through a 5-µm membrane filter to eliminate mycelia and sporangia. Finally, suppressors of the Δ*asfR* strain were obtained by single-colony isolation and phenotypic analysis of each strain. A portion of the filtered suspension was inoculated onto nutrient-rich yeast extract-beef extract-NZ amine-maltose monohydrate (YBNM) agar and cultivated at 30 °C for 2 days. Single colonies were picked up and streaked on YBNM agar, and the plates were incubated at 30 °C for 3 days. Each strain was inoculated in peptone-yeast extract-MgCl_2_ (PYM) liquid broth and cultivated with shaking at 30 °C for 2 days. After washing with 0.75% NaCl solution, the mycelia were inoculated onto HAT agar and cultivated at 30 °C for 7 days for sporangium formation. Sporangium dehiscence was analysed by suspending and incubating the sporangia harvested from the HAT agar surface in 25 mM histidine solution. Strains in which sporangia released spores under sporangium dehiscence-inducing conditions, as determined by phase-contrast microscopy, were isolated, and the spores released from the sporangia of each strain were quantified as described previously [29].

### Genome sequencing of the isolated strains

The strains isolated using the above-mentioned enrichment strategy were inoculated into PYM liquid broth and cultivated at 30 °C for 2 days. Genomic DNA was extracted using the cetyltrimethylammonium bromide method [22]. Sequencing libraries were prepared using 3 µg of DNA as the starting material, and sequencing was performed using a NovaSeq 6000 sequencer (Illumina, CA, USA). Library construction and sequencing were performed by Novogene (Beijing, China). Sequencing reads were filtered by sequence quality and mapped to the *A. missouriensis* Δ*asfR* genome sequence using the CLC Genomics Workbench (Illumina).

### Construction of the *sspA* null mutant (Δ*sspA*) strain

The upstream and downstream regions (~2 kbp each) of *sspA* were amplified using PCR. The amplified DNA fragments were digested with the appropriate restriction enzymes (Table S1) and cloned into pUC19, which was also digested with the same restriction enzymes. The generated plasmids were sequenced to confirm the absence of PCR-derived errors. The cloned fragments were digested with restriction enzymes and cloned together into pK19mob*sacB* [18,30] digested with restriction enzymes. The generated plasmid was introduced into the *A. missouriensis* wild-type strain by conjugation, as described previously [25]. Apramycin-resistant colonies resulting from single-crossover recombinations were isolated. One of them was grown in PYM liquid broth at 30 °C for 48 h, and the mycelia suspended in 0.75% (w/v) NaCl solution were spread onto the Czapek-Dox broth agar medium (BD, NJ, USA) containing extra sucrose (final concentration 5%). *A. missouriensis* cells expressing *sacB*, which encodes levansucrase, from pK19mob*sacB* exhibit lethal sensitivity to sucrose. After incubation at 30 °C for 5 days, sucrose-resistant colonies were inoculated on YBNM agar medium with or without apramycin to isolate apramycin-sensitive colonies resulting from the second single-crossover recombination as candidates for gene deletion strains. Disruption of *sspA* was confirmed using PCR.

### Construction of strains for gene complementation testing

The 3.7-, 1.7-, 1.6-, 2.3-, 1.6-, 2.8-, 3.0-, 1.2-, 1.4- and 2.0-kbp DNA fragments containing the promoter and coding sequences of *AMIS_2200*, *AMIS_42640*, *AMIS_47100*, *AMIS_47750*, *AMIS_48090*, *AMIS_53110*, *AMIS_60090*, *sspA*, *AMIS_69690* and *AMIS_79540*, respectively, were amplified using PCR. The amplified fragments were digested with *Eco*RI and *Hin*dIII and cloned into pUC19, which was also digested with the same restriction enzymes. The generated plasmids were sequenced to confirm the absence of PCR-derived errors. The cloned fragments were digested with *Eco*RI and *Hin*dIII and cloned into pTYM19-Apra, which was also digested with the same restriction enzymes. To construct the *sspA-HiBiT* gene, the HiBiT tag-coding sequence was inserted immediately upstream of the sequence encoding the C-terminal pentapeptide signal in *sspA* by overlap extension PCR. The amplified DNA fragment was digested with *Eco*RI and *Hin*dIII and cloned into pUC19, which was also digested with the same restriction enzymes. The generated plasmid was sequenced to confirm that no PCR-derived errors were present. The cloned fragment was digested with *Eco*RI and *Hin*dIII and cloned into pTYM19-Apra digested with the same restriction enzymes. The generated plasmids were introduced into the S-1, S-2 or Δ*sspA* strains by conjugation, as described previously [25]. Plasmid pTYM19-Apra was also introduced into the wild-type and Δ*sspA* strains to generate vector control strains. Apramycin-resistant colonies were then isolated.

### Extracellular detection of SspA-HiBiT

The Δ*sspA* strains harbouring pTYM19-Apra plasmids carrying *sspA* or *sspA-HiBiT* were cultivated on HAT agar at 30 °C for 7 days for sporangium formation. Then, 25 mM NH_4_HCO_3_ solution (10 ml) was poured onto the HAT agar plate and incubated at room temperature for 1 h to induce sporangium dehiscence. The zoospore suspension was collected from the surface of the HAT agar and filtered through a 5-µm membrane filter to eliminate mycelia and sporangia. The spores were collected by centrifugation and washed with 0.75% (w/v) NaCl solution and resuspended in the same solution (200 µl). The detection reagent (10 µl) of the Nano-Glo^®^ HiBiT Extracellular Detection System (Promega, WI, USA), which contained luciferin and a truncated luciferase that becomes active only in the presence of the HiBiT tag peptide, was added to a portion of the zoospore suspension (10 µl) and incubated at room temperature for 10 min in a 1.5-ml microtube. Then, luminescence from the half volume (10 µl) of the mixture was detected using an ImageQuant™ LAS 4010 system (Cytiva, Tokyo, Japan). After detection, the mixture was observed using phase-contrast microscopy to confirm that the zoospores did not lyse after incubation with the detection reagent.

### Heat resistance assay

WT and Δ*sspA* strains, both of which harboured the empty vector pTYM19-Apra, and the Δ*sspA* strain harbouring the *sspA* complementation plasmid were cultivated on HAT agar at 30 °C for 7 days for sporangium formation. Then, 25 mM NH_4_HCO_3_ solution was poured onto the HAT agar and incubated at room temperature for 1 h to induce sporangium dehiscence. The zoospore suspension was collected from the surface of the HAT agar and filtered through a 5-µm membrane filter to eliminate mycelia and sporangia. The zoospore-containing filtrate was then incubated at 50 °C in a water bath for 30 min. Every 10 min, a portion of the suspension was retrieved and transferred to a new tube in ice-cold water. The samples were diluted with 0.75% (w/v) NaCl solution, inoculated onto YBNM agar and incubated at 30 °C for 2 days. A portion of the suspension before incubation at 50 °C was inoculated onto YBNM agar in parallel. The number of zoospores that survived heat stress was estimated from the number of colonies formed on YBNM agar.

## Results

### Two missense mutations in *sspA* lead to spore release from the Δ*asfR* sporangia

**Fig. 1.**
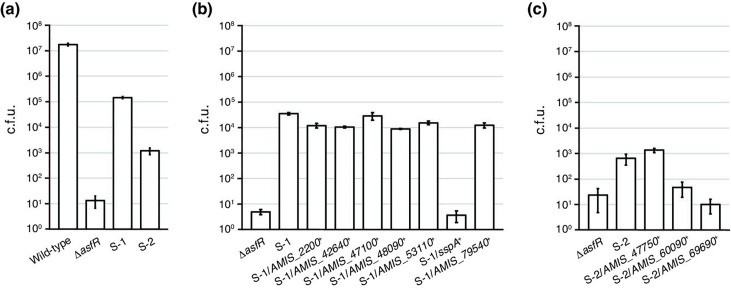
Number of spores released from the sporangia. (a) Number of spores released from sporangia of the wild-type, Δ*asfR* and two suppressor (S-1 and S-2) strains. (b) Number of spores released from sporangia of the Δ*asfR* and mutant S-1 strains and mutant S-1 harbouring each of the seven (*AMIS_2200*, *AMIS_42640*, *AMIS_47100*, *AMIS_48090*, *AMIS_53110*, *sspA* and *AMIS_79540*) complementation plasmids. (c) Number of spores released from sporangia of the Δ*asfR* and mutant S-2 strains and mutant S-2 harbouring each of the three (*AMIS_47750*, *AMIS_60090* and *AMIS_69690*) complementation plasmids. In (a)–(c), all strains were cultivated on HAT agar at 30°C for 7 days. Sporangium dehiscence was induced by pouring 25 mM NH_4_HCO_3_ solution on a sporangium-forming HAT agar plate, followed by incubation at room temperature for 1 h. The solution was retrieved from the agar surface and filtered through a 5-μm membrane filter to eliminate mycelia and sporangia. A portion of the zoospore suspension was cultivated on YBNM agar at 30°C for 2 days, and the number of colonies was counted to estimate the colony-forming unit (c.f.u.) value of the sample. The values represent mean ± standard error of three biological replicates.

To isolate suppressors of the Δ*asfR* strain, we generated a mutant library of Δ*asfR* zoospores by UV irradiation. Under laboratory conditions, sporangium dehiscence can be induced either (i) by pouring 25 mM NH_4_HCO_3_ solution on sporangia formed on HAT agar, followed by incubation for 1 h, or (ii) by suspending the sporangia harvested from the HAT agar surface in 25 mM histidine solution and incubating the suspension for 1 h. Because the number of spores released from the Δ*asfR* sporangia using method (i) was extremely low compared to that from the wild-type sporangia (sporangia of the wild-type and Δ*asfR* strains released 10^7^ and 10^1^ spores, respectively, per plate, Fig. 1a), we suspended sporangia of the Δ*asfR* strain harvested from sporangium-forming HAT agar plates in 25 mM histidine solution and vigorously mixed the suspension with glass beads to forcibly induce spore release. We generated a mutant library using the released spores, enriched the suppressors of the Δ*asfR* strain and isolated single colonies, as described in the ‘Methods’ section. We cultivated each strain on HAT agar for sporangium formation and quantified the spores released from sporangia using method (i) by counting the colonies formed on YBNM agar after incubation at 30 °C for 2 days. In this experiment, all colonies on YBNM agar were formed from the spores released from sporangia, because the zoospore suspension retrieved from the HAT agar surface was filtered through a 5-µm membrane filter to eliminate mycelia and sporangia. We obtained two suppressor strains, designated as S-1 and S-2, whose sporangia released larger numbers of spores than those from Δ*asfR* sporangia; the sporangia of mutants S-1 and S-2 released 10^5^ and 10^3^ spores, respectively, per HAT agar plate (Fig. 1a).

To identify the mutations generated, we determined the genome sequences of two suppressor strains. Among the obtained data, we identified 11 single-nucleotide variants (SNVs) within 9 genes and 3 SNVs plus 1 multiple-nucleotide variant within 4 genes, which changed the amino acid sequence of gene products, in mutants S-1 and S-2, respectively (Table S2). To determine the mutations responsible for the release of spores in the suppressor strains, we conducted a gene complementation test, in which the wild-type allele of the mutated genes was sequentially introduced into the suppressor strains using the chromosome-integrating vector pTYM19-Apra [27,31]. In this experiment, seven of the nine candidate genes (*AMIS_2200*, *AMIS_42640*, *AMIS_47100*, *AMIS_48090*, *AMIS_53110*, *AMIS_68180* (*sspA*) and *AMIS_79540*) were introduced individually into mutant S-1, and three of the four candidate genes (*AMIS_47750*, *AMIS_60090* and *AMIS_69690*) were introduced individually into mutant S-2. We quantified the spores released from the sporangia of the suppressor strains harbouring the complementation plasmids, together with the Δ*asfR* and suppressor strains used as controls. The number of spores released from the sporangia of mutant S-1 significantly decreased following the introduction of the *sspA* complementation plasmid and that from the sporangia of mutant S-2 decreased following the introduction of the *AMIS_60090* or *AMIS_69690* complementation plasmid (Fig. 1b, c). These results suggested that the mutation in *sspA* was responsible for the release of spores from the mutant S-1 sporangia and that the mutations in *AMIS_60090* and *AMIS_69690* were involved in the release of spores from the mutant S-2 sporangia. Hereafter, we focused on *sspA* because the number of spores released from the mutant S-1 sporangia decreased to almost the same level as that from Δ*asfR* sporangia by the introduction of *sspA* (Fig. 1b).

In mutant S-1, two SNVs within *sspA* replaced Gly-179 with Glu and Gly-181 with Ser in the 191 aa product (Table S2 and Fig. S1). The SignalP 6.0 server (https://services.healthtech.dtu.dk/services/SignalP-6.0/) predicted that SspA has a signal peptide for the general secretory pathway (the predicted cleavage site is between residues 44 and 45). A protein database search using InterPro version 101.0 (https://www.ebi.ac.uk/interpro/) showed that the C-terminal portion of SspA harbours a sortase-dependent cell wall-sorting signal (accession number IPR019931, residues 158–191), which includes Gly-179 and Gly-181 that are substituted in mutant S-1 (Fig. S1). In addition, the mature part of SspA was predicted to be disordered using AlphaFold 2 ColabFold version (https://colab.research.google.com/github/sokrypton/ColabFold/blob/main/AlphaFold2.ipynb) (Fig. S2). The entire mature part of SspA was also predicted to be disordered by MobiDB-lite (http://old.protein.bio.unipd.it/mobidblite/) [32] (Fig. S1). No other conserved domains or motifs were identified in SspA. Thus, these *in silico* analyses suggested that SspA is an intrinsically disordered, cell surface-displayed protein.

### Transcription profile of *sspA* throughout the *A*. *missouriensis* life cycle

Previously, we performed exhaustive RNA sequencing analysis at various time points during the life cycle of *A. missouriensis* [16]. In this analysis, RNA samples for vegetative growth were prepared from substrate hyphae grown on YBNM agar for 1 day in triplicate. For sporangium formation, RNA samples were prepared from mycelia and/or sporangia grown on HAT agar for 1, 3, 6 and 15 days, in triplicate at each time point. For sporangium dehiscence, RNA samples were prepared from sporangia (including some substrate hyphae) suspended and incubated in 25 mM histidine solution for 0, 15 and 60 min in triplicate at each time point. According to this analysis, the transcript levels of *sspA* were highly upregulated at the early stages of sporangium formation, whereas they were gradually downregulated at later stages of sporangium formation (Fig. S3).

 In *A. missouriensis*, three FliA-family sigma factors, FliA1, FliA2 and FliA3, are involved in sporangium formation, spore dormancy and sporangium dehiscence [25]. In our previous study, the transcriptomes of the wild-type strain and null mutant strains of *fliA1* (Δ*fliA1*), *fliA2* (Δ*fliA2*) and *fliA3* (Δ*fliA3*) were analysed using RNA samples extracted from cells cultivated on HAT agar at 30 °C for 6 days [25]. According to this analysis, *sspA* transcription was 2.5-, 2.5- and 10.2-fold upregulated in the Δ*fliA1*, Δ*fliA2* and Δ*fliA3* strains, respectively, compared to the wild-type strain, although transcriptional regulation of *sspA* awaits further investigation.

### SspA plays a pivotal role in the formation of structurally rigid sporangia

**Fig. 2.**
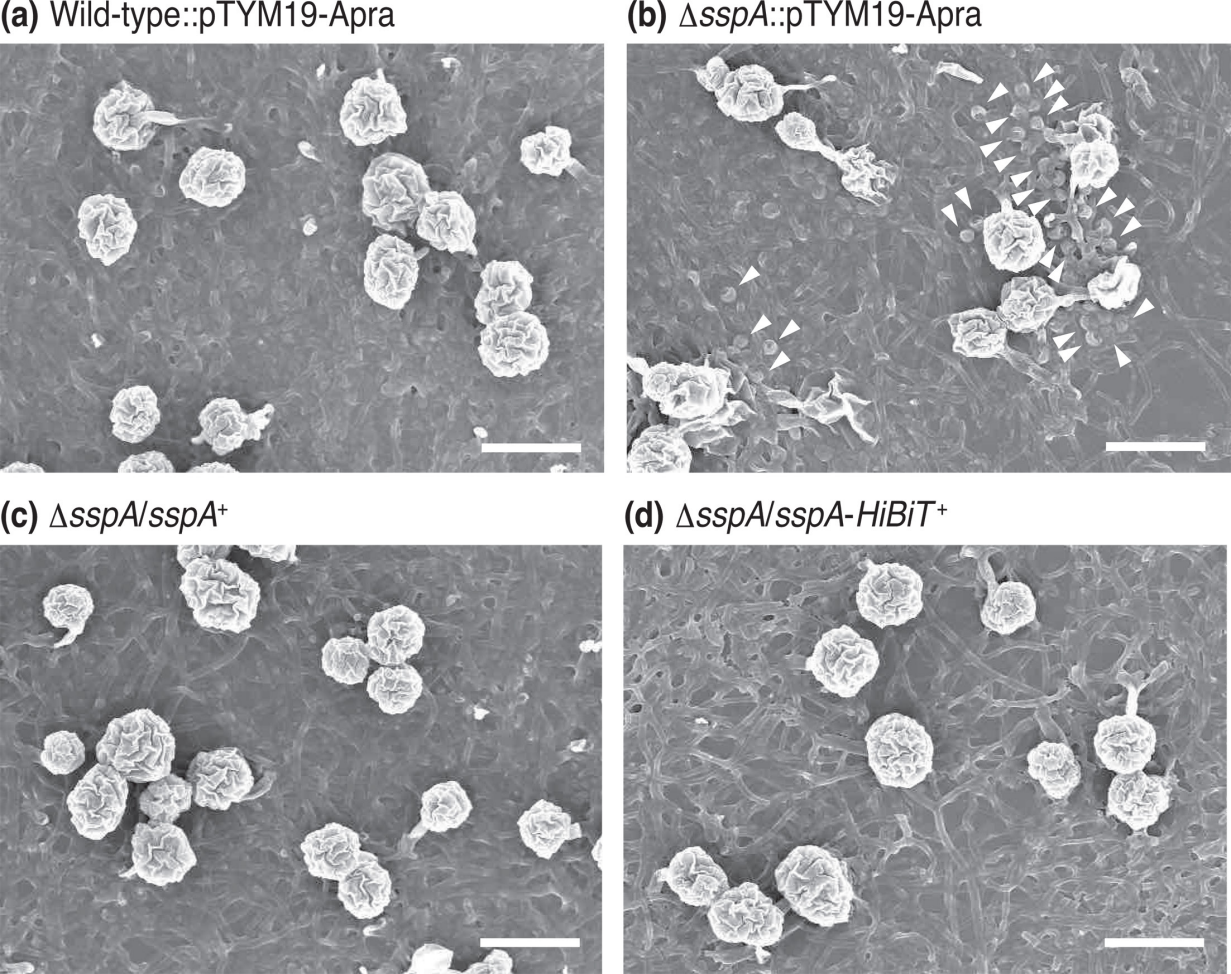
SEM observation of sporangia produced on HAT agar after 7 days of cultivation. (**a**) Wild-type strain harbouring pTYM19-Apra. (**b**) Δ*sspA* strain harbouring pTYM19-Apra. Naked spores are indicated by arrowheads. (**c**) Δ*sspA* strain harbouring the *sspA* complementation plasmid. (**d**) Δ*sspA* strain harbouring the *sspA-HiBiT* complementation plasmid. In (**a**), (**c**) and (**d**), naked spores were not observed. HiBiT is an 11-aa peptide tag, and the HiBiT tag-coding sequence was inserted immediately upstream of the sequence encoding the C-terminal pentapeptide signal in *sspA* (see Fig. S1). Scale bars, 5 µm.

To examine the *in vivo* functions of SspA, we generated a Δ*sspA* strain. No differences were observed between the wild-type and Δ*sspA* strains by macroscopic observation of mycelia or sporangia formed on YBNM and HAT agars. To examine sporangium formation in detail, we observed the mycelia and sporangia of both strains grown on HAT agar at 30 °C for 7 days using SEM. In this experiment, the wild-type and Δ*sspA* strains harboured pTYM19-Apra for the gene complementation test described below. The wild-type strain produced globose or subglobose sporangia with short sporangiophores (Fig. 2a). In the Δ*sspA* strain, sporangia of normal shape were produced, but abnormal sporangia with squashed shapes were also observed (Fig. 2b). Around the squashed sporangia, naked spores were observed, which appeared to have been artificially released from the squashed sporangia during SEM sample preparation (Fig. 2b). In a gene complementation test, the introduction of pTYM19-Apra harbouring *sspA* with its own promoter into the Δ*sspA* strain restored normal sporangium formation (Fig. 2c). Notably, no naked spores were observed in the wild-type and *sspA* complementation strains under the same experimental conditions (Fig. 2a, c).

**Fig. 3.**
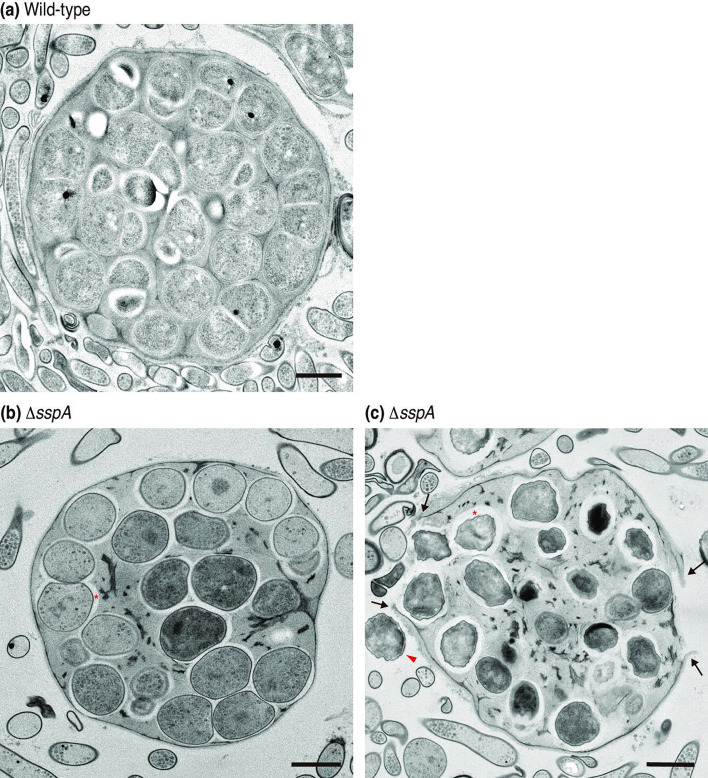
TEM observation of ultrathin sections of sporangia produced on HAT agar. (a) Wild-type strain. (b, c) Δ*sspA* strain. In (b) and (c), the peripheral region of each spore has low electron density, and one of the electron-lucent regions is indicated by a red asterisk in each panel. In (c), a probable released spore is indicated by a red arrowhead, and the breakpoints of the sporangium outer envelope are indicated by arrows. Scale bars, 1 μm.

Next, to examine sporangiospore maturation inside sporangia, ultrathin sections of the wild-type and Δ*sspA* strains, both of which were cultivated under identical conditions used for SEM, were subjected to TEM. In the wild-type sporangia, round spores of similar sizes surrounded by the sporangium matrix were observed (Fig. 3a). In the Δ*sspA* sporangia, although round spores were observed, the space between spores inside a sporangium was larger than that in the wild-type sporangia (Fig. 3b). Although the sporangium matrix appeared to be produced in the space between spores, the electron density at the peripheral region of each spore was quite low (Fig. 3b). In another sporangium of the Δ*sspA* strain, spores with round and oval shapes of various sizes were observed, and many of them had wider electron-lucent peripheral regions (Fig. 3c). The surface of the spores in this Δ*sspA* sporangium was rough, in contrast to the smooth surface of the wild-type spores (Fig. 3a, c), and the sporangium outer envelope was damaged (Fig. 3c), which might facilitate the release of spores outside the sporangium, as observed by SEM (Fig. 2b).

**Fig. 4.**
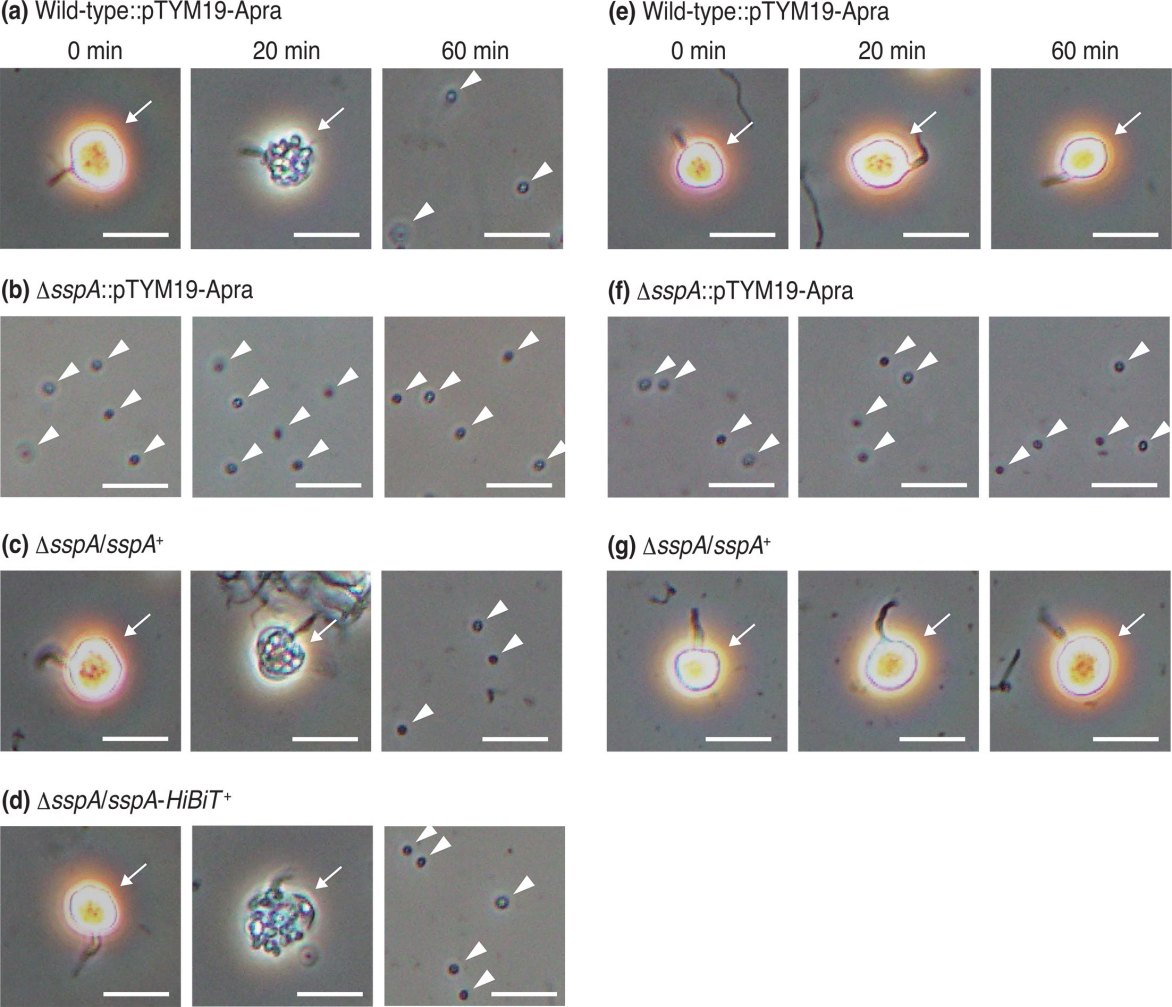
Observation of sporangia and mycelia in aquatic environments using phase-contrast microscopy. Sporangia and mycelia produced on HAT agar were harvested and suspended in 25 mM histidine (a–d) or 50 mM NaCl (e–g) solutions. Micrographs of the wild-type (**a, e**) and Δ*sspA* (**b, f**) strains, both of which harboured the empty vector pTYM19-Apra, and the Δ*sspA* strain harbouring the *sspA* (**c, g**) or *sspA-HiBiT* (**d**) complementation plasmid are shown. Left panels were obtained immediately after suspension. The middle panels were obtained 20 min after suspension. Right panels were obtained 60 min after suspension. Sporangia (including those whose membrane became transparent) and released spores are indicated by arrows and arrowheads, respectively. Scale bars, 10 µm.

We examined sporangium dehiscence and zoospore motility using phase-contrast microscopy. In this experiment, sporangia and mycelia formed on HAT agar were harvested and suspended in 25 mM histidine solution to induce sporangium dehiscence. Under these conditions, the wild-type sporangia appeared phase-bright immediately after suspension in histidine solution, and the sporangium outer envelope gradually became transparent before spore release (Fig. 4a). In contrast, the process was markedly different in the Δ*sspA* strain; most sporangia collapsed and released spores into the external environment immediately after suspension in histidine solution (Fig. 4b). Considering the observations by SEM and TEM (Figs2  3), we assume that the sporangium structures of the Δ*sspA* strain are highly fragile and easily collapse in aquatic solutions. To examine this assumption, sporangia and mycelia of the wild-type and Δ*sspA* strains grown on HAT agar were harvested and suspended in 50 mM NaCl solution, followed by incubation at room temperature for 1 h. In this solution, sporangium dehiscence of the wild-type strain is not induced [33]. The wild-type sporangia remained phase-bright during incubation (Fig. 4e). In contrast, similar to the process in 25 mM histidine solution, the Δ*sspA* sporangia collapsed and released spores into the external environment immediately after suspension (Fig. 4f). The phenotypic changes observed in the Δ*sspA* strain were restored by introducing the *sspA* complementation plasmid (Fig. 4c, g).

**Fig. 5.**
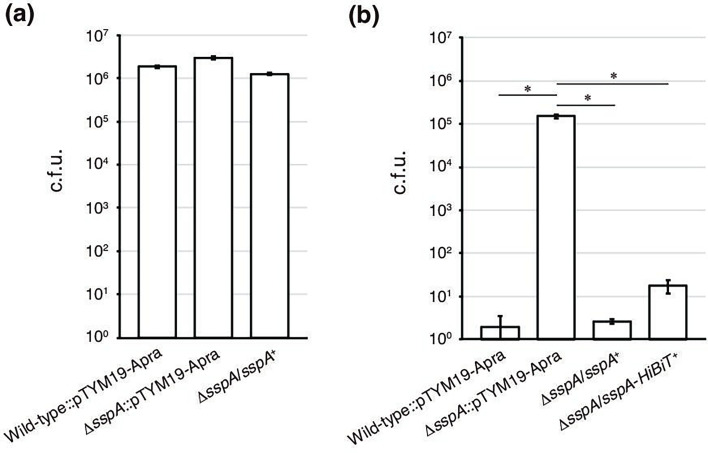
Number of spores released from the sporangia. The wild-type and Δ*sspA* strains, both of which harboured the empty vector pTYM19-Apra, and the Δ*sspA* strain harbouring the *sspA* complementation plasmid were cultivated on HAT agar at 30°C for 7 days. Then, 25 mM NH_4_HCO_3_ solution (a) or 50 mM NaCl solution (b) was poured onto a sporangium-forming HAT agar plate, followed by incubation at room temperature for 1 h. In (b), the Δ*sspA* strain harbouring the *sspA*-*HiBiT* complementation plasmid was also used. The solution was retrieved from the agar surface and filtered through a 5-μm membrane filter to eliminate mycelia and sporangia. A portion of the zoospore suspension was cultivated on YBNM agar at 30°C for 2 days, and the number of colonies was counted to estimate the c.f.u. value of the sample. The values represent mean ± standard error of three biological replicates. In (b), differences were analysed using Student’s *t*-test. *P<0.001.

To quantitatively evaluate the observations made by phase-contrast microscopy, we counted the spores released from the sporangia of the wild-type and Δ*sspA* strains. Sporangium dehiscence was induced by pouring 10 ml of 25 mM NH_4_HCO_3_ solution onto sporangia formed on a HAT agar plate, followed by incubation for 1 h (we previously found that the spore counts reached a plateau after 1 h of incubation, although a small portion of the sporangia might not open to release spores for 1 h). The number of spores released from sporangia was estimated from the number of colonies formed on YBNM agar after incubation at 30 °C for 2 days, as described previously [28]. The number of colonies formed on YBNM agar did not differ significantly between the wild-type and Δ*sspA* strains (Fig. 5a). This result indicated that the wild-type and Δ*sspA* strains produced similar numbers of viable spores. Based on the observations using phase-contrast microscopy (Fig. 4a, b), we hypothesized that the Δ*sspA* sporangia collapsed and released spores, whereas the wild-type sporangia normally opened to release spores through sporangium dehiscence. To test this assumption, we quantified the spores released from sporangia by pouring 10 ml of 50 mM NaCl solution, instead of 25 mM NH_4_HCO_3_ solution, onto a sporangium-forming HAT agar plate, because we previously found that sporangium dehiscence is inhibited in 50 mM NaCl solution. Only a few spores were released from the wild-type sporangia on one HAT agar plate (Fig. 5b). In contrast, more than 10^5^ spores (per HAT agar) were released in the Δ*sspA* strain, indicating that the Δ*sspA* sporangia collapsed without normal sporangium dehiscence (Fig. 5b). This phenotypic change was also restored by introducing the *sspA* complementation plasmid into the Δ*sspA* strain (Fig. 5b). These results clearly demonstrated that SspA is essential for the formation of rigid sporangium structures that do not easily collapse in aquatic environments.

### SspA is localized on the zoospore surface

**Fig. 6.**
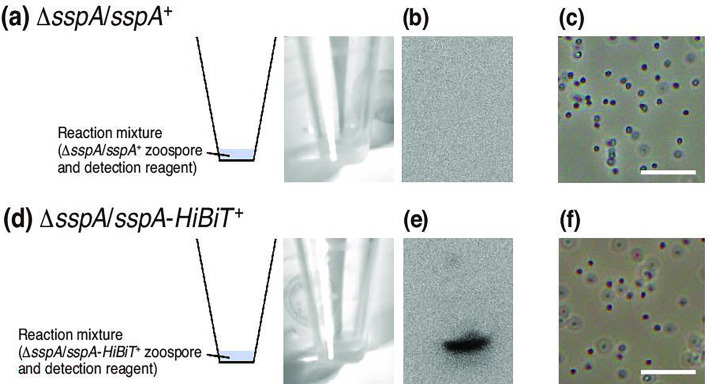
Detection of SspA-HiBiT on zoospore surface. A portion of the zoospore suspension of the Δ*sspA* strain harbouring the *sspA* (a–c) or *sspA*-*HiBiT* (d–f) complementation plasmid was incubated with the detection reagent of the Nano-Glo^®^ HiBiT Extracellular Detection System (Promega) at room temperature for 10 min in a 1.5-ml microtube. The 1.5-ml microtube containing 10 μl of each reaction mixture was illuminated with light, and luminescence was detected using an ImageQuant™ LAS 4010 image analyser (Cytiva) (b, e). Panels (a) and (d) show photographs taken by the image analyser and their illustrations. The zoospores were observed using phase-contrast microscopy after detection to confirm that they were not subjected to lysis (c, f). In (c) and (f), scale bars, 10 mm. See the ‘Methods’ section for a detailed procedure.

As described above, SspA has a sortase-dependent cell wall-localizing signal in its C-terminal region (Fig. S1). Thus, we examined the localization of SspA using HiBiT technology (Promega). In this experiment, we constructed a mutated copy of *sspA* encoding the HiBiT-tagged SspA protein (SspA-HiBiT) by inserting a nucleotide sequence coding for the 11-aa HiBiT tag immediately upstream of the C-terminal pentapeptide motif (Fig. S1). We generated a plasmid containing *sspA-HiBiT* with its native promoter and introduced this plasmid into the Δ*sspA* strain. After cultivation on HAT agar for 7 days, the Δ*sspA* strain harbouring the *sspA-HiBiT*-expressing plasmid formed sporangia with normal shapes when observed by SEM (Fig. 2d). Sporangia of this strain opened normally to release spores when suspended in 25 mM histidine solution (Fig. 4d). We quantified the spores released from sporangia by pouring 50 mM NaCl solution on the sporangium-forming HAT agar plate. The number of spores released from the sporangia of the Δ*sspA* strain harbouring the *sspA-HiBiT*-expressing plasmid was four orders of magnitude lower than that of the Δ*sspA* strain harbouring the empty vector, indicating that SspA-HiBiT was functional (Fig. 5b). Thus, we examined whether SspA-HiBiT was localized on the surface of zoospores using the HiBiT extracellular detection reagent (Promega). The non-lytic reagent contains a luciferase substrate and LgBiT protein. The HiBiT peptide tag binds to LgBiT to form an active luciferase. While no luminescence signal was detected in the reaction sample containing zoospores of the Δ*sspA* strain harbouring *sspA*, a significant luminescence signal was detected in the reaction sample containing the zoospores of the △*sspA* strain harbouring *sspA-HiBiT*, indicating the presence of SspA-HiBiT in the extracellular space of the zoospores (Fig. 6a, b, d and e). We confirmed that the zoospores did not lyse after incubation with the detection reagent by observing them using phase-contrast microscopy (Fig. 6c, f). These results demonstrated that SspA-HiBiT was localized on the zoospore surface.

### Enhanced heat tolerance of Δ*sspA* zoospore

**Fig. 7.**
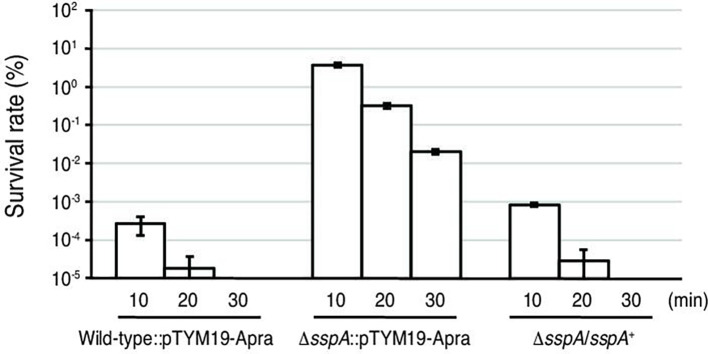
Heat resistance of the zoospores. The wild-type and Δ*sspA* strains, both of which harboured the empty vector pTYM19-Apra, and the Δ*sspA* strain harbouring the *sspA* complementation plasmid were cultivated on HAT agar at 30°C for 7 days. Sporangium dehiscence was induced by pouring 25 mM NH_4_HCO_3_ solution on a sporangium-forming HAT agar plate, followed by incubation at room temperature for 1 h. The solution was retrieved from the agar surface and filtered through a 5-μm membrane filter to eliminate mycelia and sporangia. Suspensions of zoospores were incubated at 50°C for 30 min. Aliquots of the suspensions were sampled before and 10, 20 and 30 min after the onset of incubation. A portion of the sample was cultivated on YBNM agar at 30°C for 2 days, and the number of colonies was counted to estimate the c.f.u. value of the sample. The heat resistance of zoospores was determined at the survival rate by taking the c.f.u. before incubation at 50°C as 100% in each suspension. The values represent mean ± standard error of three biological replicates.

Because SspA was detected on the zoospore surface, we hypothesized that the loss of SspA affects the stress tolerance of zoospores. In *Streptomyces*, dormant spores are typically resistant to moderately high temperatures. Thus, heat sensitivity is a hallmark of spore cell wall defects [34,35]. To investigate the heat tolerance of zoospores, we prepared the zoospores of the wild-type and Δ*sspA* strains by pouring 25 mM NH_4_HCO_3_ solution onto sporangium-forming HAT agar plates and filtering through a 5-µm membrane filter, followed by incubation at 50 °C for 30 min. Viable zoospores were estimated every 10 min after heat treatment by forming colonies on YBNM agar. Unexpectedly, the survival rate of the Δ*sspA* zoospores was higher than that of the wild-type zoospores, indicating that the Δ*sspA* zoospores were more heat-resistant than the wild-type zoospores (Fig. 7). In a gene complementation test, zoospores of the Δ*sspA* strain harbouring the *sspA* complementation plasmid formed a similar number of colonies as the wild-type strain (Fig. 7).

## Discussion

In this study, we revealed that SspA plays a pivotal role in the formation of rigid sporangium structures in *A. missouriensis*. Although we have identified more than ten transcriptional regulatory proteins, including transcriptional regulators and alternative sigma factors, and several enzymes as key proteins involved in morphological development in *A. missouriensis*, SspA is the first example of a structural protein required for sporangium formation. We assume that SspA is produced in extending hyphae inside a sporangium and anchored to the cell wall peptidoglycan by the function of sortase during sporangiospore maturation in *A. missouriensis*. In the *A. missouriensis* genome, one (*AMIS_500*) and four (*AMIS_11000*, *AMIS_17370*, *AMIS_33280* and *AMIS_36430*) genes encode putative class E and class F sortases, respectively. Characterization of these sortase candidates indicated that the class E sortase (AMIS_500) is responsible for the cell-wall localization of SspA (the manuscript will be published elsewhere).

In *Staphylococcus aureus*, sortase-dependent surface proteins are covalently anchored to the pentaglycine cross-bridge of the peptidoglycan precursor [7,36]. Peptidoglycan of the genus *Micromonospora* has been reported to have the peptide subunit glycyl-d-glutaminyl-*meso*-diaminopimelyl-(d-alanine), which is directly cross-linked between the carboxy group of d-alanine in one peptide subunit and the ω-amino group of *meso*-diaminopimelic acid in another peptide subunit [37]. The C-terminal d-alanine is occasionally eliminated by d-alanine carboxypeptidase [37]. Considering that the genera *Micromonospora* and *Actinoplanes* belong to the family *Micromonosporaceae*, the peptidoglycan of the genus *Actinoplanes* is expected to have the same peptide subunit and cross-linking. In this case, SspA seems to be attached to the peptidoglycan precursor through isopeptide bond formation between the carboxy group of the C-terminal threonine residue in SspA and the ω-amino group of *meso*-diaminopimelic acid in the peptidoglycan precursor.

In the Δ*sspA* strain, the sporangium matrix was not produced normally, and the sporangia easily collapsed in aquatic environments. Although the Δ*sspA* sporangia were fragile, zoospores released from the sporangia swam in aquatic environments and germinated in suitable niches, similar to wild-type zoospores. Thus, SspA is more relevant to sporangium formation than to zoospore biology, despite its localization on the spore surface. In this respect, the evolution of SspA seems to be closely related to the ability of *A. missouriensis* to form sporangia, in which appendages of spores, such as SspA, play key roles in constructing unusual prokaryotic multicellular structures. SspA homologues are conserved among the genera *Couchioplanes*, *Paractinoplanes*, *Symbioplanes*, *Jidongwangia*, *Winogradskya* and *Mangrovihabitans*, all of which belong to the family *Micromonosporaceae*, along with the genus *Actinoplanes*.

Recently, we reported two glycosylhydrolases, GimA and GimB, which are required for the degradation of the sporangium matrix [38]. In the double disruptant of *gimA* and *gimB*, spores were captured by the sporangium matrix and not released, despite the degradation of the sporangium outer envelope under sporangium dehiscence-inducing conditions [38]. In contrast, Δ*sspA* sporangia easily collapsed in solution and released spores. These observations indicate that Δ*sspA* sporangia cannot produce a normal sporangium matrix that tightly holds spores when the sporangium outer envelope is broken. TEM analysis of the Δ*sspA* sporangia revealed that the electron density was very low in the peripheral region of each spore, indicating that a normal sporangium matrix was not produced and/or preserved, at least in this region. How is SspA involved in the production and/or preservation of the sporangium matrix in sporangia? We recently indicated that a major component of the sporangium matrix is a polysaccharide consisting of repeating oligosaccharides and that GimA and GimB digest this polysaccharide [38]. We also identified a gene cluster whose gene products provide a Wzx/Wzy-dependent pathway that is responsible for sporangium matrix polysaccharide biosynthesis. In this pathway, oligosaccharide repeating units are assembled by glycosyltransferases inside the cell and translocated across the cytoplasmic membrane by the Wzx protein. Subsequently, polymerization of the repeating units occurs on the outer leaflet of the membrane by the Wzy protein [39]. Several intrinsically disordered proteins form rigid structures upon interaction with partner proteins or molecules via coupled folding and binding [40,42]. Because SspA is predicted to be an intrinsically disordered protein (Figs S1 and S2), SspA may support the polymerization of the repeating units via interaction with the Wzy enzyme. Alternatively, SspA may function as a foothold for the secretion of the sporangium matrix polysaccharide through the cell wall via its interaction with the polysaccharides. Furthermore, SspA may preserve the polysaccharide around spores. In another scenario, SspA may contribute to the level of cross-linking of cell wall peptidoglycan by competing with or facilitating covalent bond formation between peptidoglycan side chains. Indeed, Δ*sspA* zoospores showed higher heat resistance than wild-type zoospores, suggesting that the level of cross-linking in the cell wall was altered (Fig. 7). We speculate that SspA provides an extracellular space suitable for polymerization and/or secretion to the sporangium matrix component by altering the frequency of peptidoglycan cross-linking. In any case, loss of SspA in the Δ*sspA* strain induced severe defects in the production and/or preservation of the sporangium matrix. Deficiency in normal sporangium matrix formation seems to reduce the strength of the sporangium outer envelope.

Initially, we attempted to identify the target protein(s) of AsfR by isolating suppressors of the Δ*asfR* strain because AsfR is likely to bind to other protein(s) to modulate its function [20]. However, we failed to obtain experimental evidence supporting the relationship between AsfR and SspA. Considering the phenotypic changes in the Δ*sspA* strain, strain S-1 seems to have been isolated because of its fragile structure of sporangia, and SspA does not seem to be involved in the function of AsfR. Thus, our initial plan was not successful, but we identified SspA as an essential structural protein for the production and/or secretion of sporangium matrix polysaccharides. Therefore, this study provides important insights into the molecular mechanisms underlying sporangium formation in *A. missouriensis*.

## Supplementary material

10.1099/mic.0.001656 Uncited Supplementary Material 1.

## Abbreviations

c.f.u.: colony-forming unit
HAT: humic acid-trace element
PYM: peptone-yeast extract-MgCl_2_
SEM: scanning electron microscopy
SNVs: single-nucleotide variants
TEM: transmission electron microscopy
YBNM: yeast extract-beef extract-NZ amine-maltose monohydrate
