# Supplementary material for: Lack of a putative intrinsically disordered protein anchored to the sporangiospore cell wall causes fragile sporangium formation in Actinoplanes missouriensis
Source: Microbiology (Reading). 2026 Jan 14;172(1):001656. doi: 10.1099/mic.0.001656 (PMC13293333; doi:10.1099/mic.0.001656)
Supplement: Uncited Supplementary Material 1. [file mic-172-01656-s001.pdf]

Supporting Information for

Lack of a putative intrinsically disordered protein anchored to the sporangiospore cell wall causes fragile sporangium formation in *Actinoplanes missouriensis*

Zhuwen Tan,[1] Takeaki Tezuka,[1,2] Yasuo Ohnishi[1,2]

[1]Department of Biotechnology, Graduate School of Agricultural and Life Sciences, The University of Tokyo, Bunkyo-ku, Tokyo, Japan

[2]Collaborative Research Institute for Innovative Microbiology, The University of Tokyo, Bunkyo-ku, Tokyo, Japan

This PDF file includes:

Figures S1 to S3
Tables S1 and S2

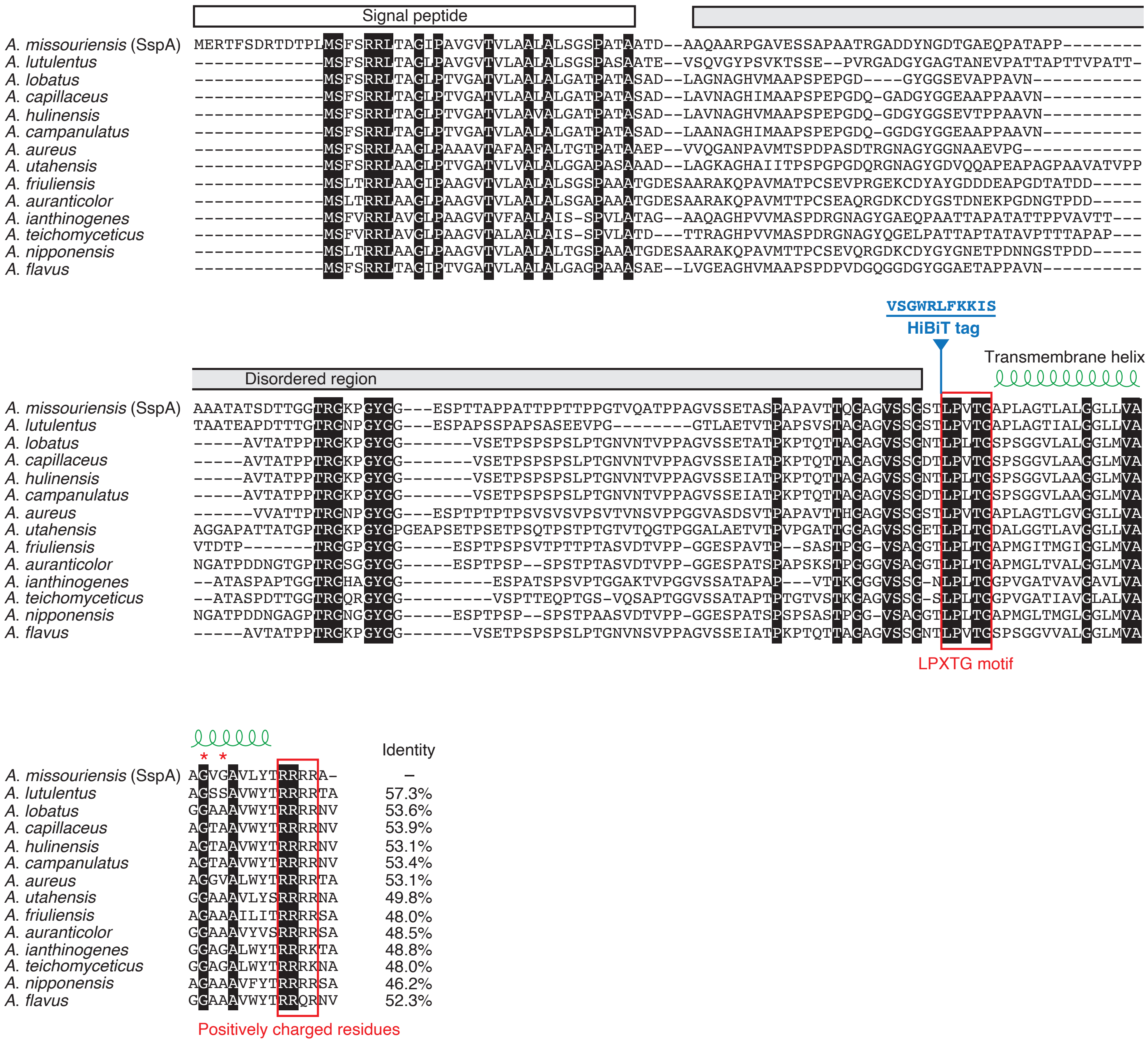

Fig. S1. Amino acid sequence alignment of SspA and its homologs from 14 *Actinoplanes* bacteria, including *A. missouriensis*. Identical amino acid residues are shown in black background. The predicted signal peptide and intrinsically disordered region in SspA are shown above the alignment. The LPXTG motif and positively charged residues of the sortase recognition sequence are indicated by red rectangles. The predicted transmembrane helix in SspA and the location of the HiBiT tag in SspA-HiBiT are shown above the alignment. The two Gly residues in SspA mutated in strain S-1 are indicated by red asterisks. The similarity between the amino acid sequence of each protein and that of SspA is shown at the end of the sequence. All sequences exhibit a similarity of at least 46.2% identity.

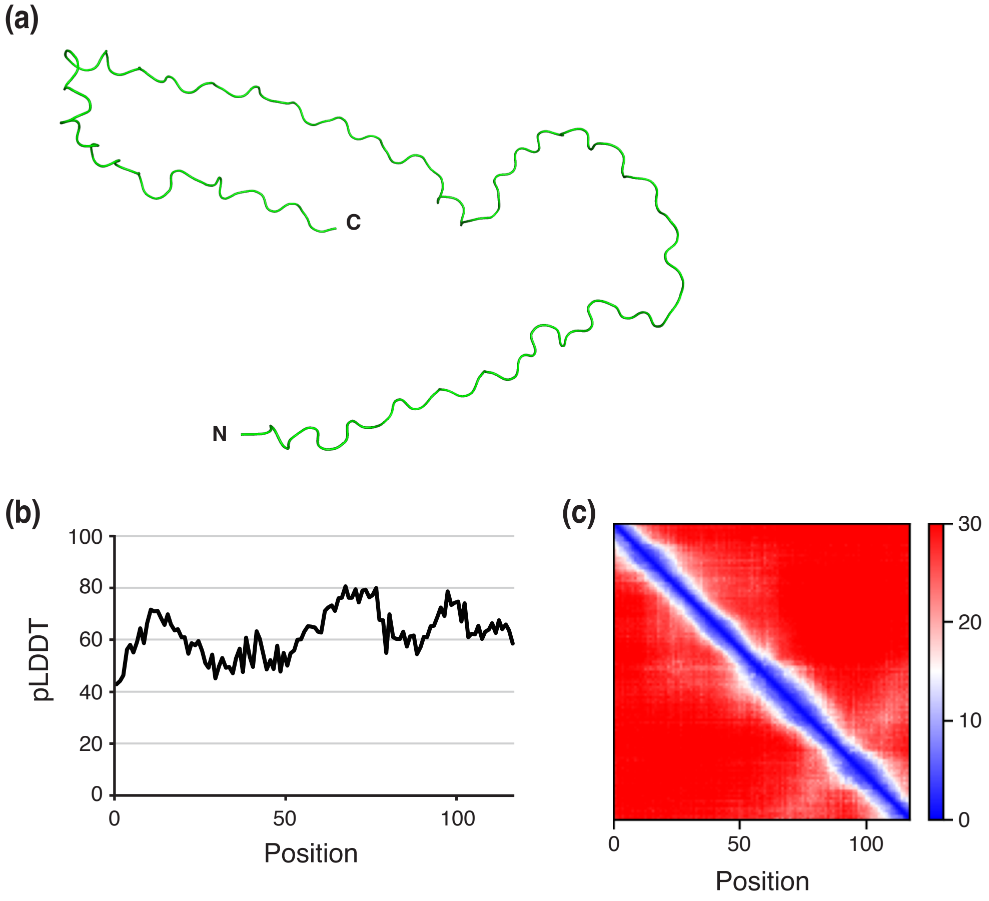

Fig. S2. AlphaFold 2-based prediction of SspA structure. (a) Predicted structure of mature SspA. The polypeptide is shown by ribbon representation and is colored green. (b) Predicted local distance difference test (pLDDT) score. The pLDDT score, which ranges from 0 to 100, characterizes local structural accuracy. (c) Predicted aligned error (PAE) score. The PAE score corresponds to the topological accuracy between each residue.

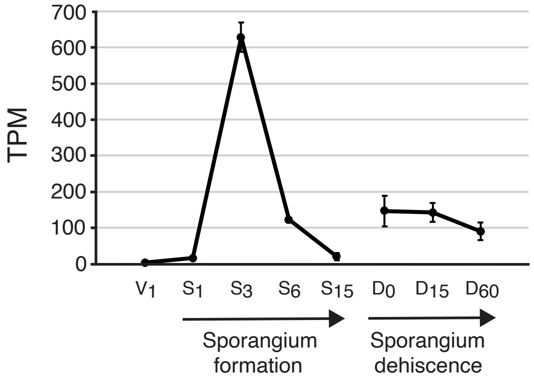

Fig. S3. Transcript levels of *sspA*. Transcripts were examined using RNA-Seq analysis under various culture conditions. RNA samples were prepared from substrate hyphae grown on YBNM agar for 1 day (V_1), substrate hyphae or mixtures of substrate hyphae and sporangia grown on HAT agar for 1, 3, 6, and 15 days (S_1, S_3, S_6, and S_{15}, respectively), and sporangia (including some substrate hyphae) incubated in 25 mM histidine solution to induce sporangium dehiscence for 0, 15, and 60 min (D_0, D_{15}, and D_{60}, respectively). The average number of transcripts per million mapped reads (TPM) values ± standard errors from three biological replicates are shown.

Table S1. Primers used in this study

Primer name	Sequence (5' to 3')[a]	Restriction enzyme	Used for
b10-AMIS_47750-SF2	GGAATTCGTACCCCTGATCGTGGTGAT	EcoRI	Gene complementation
b10-AMIS_47750-SR	GCCAAGCTTTCCGGGAACGCGTAGTACAA	HindIII	Gene complementation
b10-AMIS_60090-SF2	GGAATTCGTTGGTCTGCACGCTGAACT	EcoRI	Gene complementation
b10-AMIS_60090-SR	GCCAAGCTTGTTCTGACTCACTGTCTGCT	HindIII	Gene complementation
b10-AMIS_69690-SF	GGAATTCCCTCATGAACGAGCAACTCA	EcoRI	Gene complementation
b10-AMIS_69690-SR	GCCAAGCTTTGGCATCGGGCTGTTCATCA	HindIII	Gene complementation
c7-AMIS_2200-SF1	GGAATTCAAGAACGCGCAGAACTTGCA	EcoRI	Gene complementation
c7-AMIS_2200-SR1	GCCAAGCTTTCCTCGTCGTAGGCCTCGAT	HindIII	Gene complementation
c7-AMIS_42640-SF1	GGAATTCATGGCCCGGGAAGCGATGAT	EcoRI	Gene complementation
c7-AMIS_42640-SR1	GCCAAGCTTATCCAGCAGGGACACGACCA	HindIII	Gene complementation
c7-AMIS_47100-SF1	GGAATTCATCGTCTTCGGTCTGCTCGA	EcoRI	Gene complementation
c7-AMIS_47100-SR1	GCCAAGCTTGATCACCGAGAAGATCAGCA	HindIII	Gene complementation
c7-AMIS_48090-SF1	GGAATTCCATGGTCAACACCCCGATCA	EcoRI	Gene complementation
c7-AMIS_48090-SR1	GCCAAGCTTGATCAGCGACTTCCAGACCA	HindIII	Gene complementation
c7-AMIS_53110-SF1	GGAATTCCATTCGGACCGATCCTGGTT	EcoRI	Gene complementation
c7-AMIS_53110-SR1	GCCAAGCTTCTCGATCTGCGGTCGATCGT	HindIII	Gene complementation
c7-AMIS_68180-SF1	GGAATTCGTTGATGGTGACGCTGGCCT	EcoRI	Gene complementation
c7-AMIS_68180-SR1	GCCAAGCTTGAGGTAGTACTCGAAGACGT	HindIII	Gene complementation
c7-AMIS_79540-SF1	GGAATTCCTGGCGTACAGCGATTCTGA	EcoRI	Gene complementation
c7-AMIS_79540-SR1	GCCAAGCTTATGCCGTCCATGATCGGCAT	HindIII	Gene complementation
AMIS_60090-UF1	GGAATTCCAGACCGATGTCACCACCCT	EcoRI	Gene disruption
AMIS_60090-UR1	GCTCTAGATTGAACGCCTTGAGCTCGCT	XbaI	Gene disruption
AMIS_60090-DF1	GCTCTAGACGTGGTCACCATCGAGGTCA	XbaI	Gene disruption
AMIS_60090-DR1	GCCAAGCTTACGGTGACGACCGAGCAGTT	HindIII	Gene disruption
AMIS_68180-UF2	GCTCTAGAGCTGCAGACGGTGAAGTACT	XbaI	Gene disruption
AMIS_68180-UR2	AAAACCTGCAGGGAAGCTCATGAGAGGAGTAT	Sse8387I	Gene disruption
AMIS_68180-DF2	AAAACCTGCAGGTGATCCGTCTTCTCGGATGT	Sse8387I	Gene disruption
AMIS_68180-DR1	GCCAAGCTTGACTTGAAGGTGCCTCAGGA	HindIII	Gene disruption
AMIS_69690-UF1	GGAATTCGTACTCACCGAAGTGCTGCT	EcoRI	Gene disruption
AMIS_69690-UR1	GCTCTAGAATCGGTGCTGGATGACGTCA	XbaI	Gene disruption
AMIS_69690-DF1	GCTCTAGAATGCAGGAGCTGGACGCGAT	XbaI	Gene disruption
AMIS_69690-DR1	GCCAAGCTTCGAACTCTCCGTGCATCGAT	HindIII	Gene disruption
AMIS_60090-CF	GTGGATCTTCAGGACGAACT		Colony PCR
AMIS_60090-CR	CGTGTCCAGCAGGTTGATCA		Colony PCR
AMIS_68180-CF	ATCCGTACCTCGTCGGTCTT		Colony PCR
AMIS_68180-CR	GAGGTAGTACTCGAAGACGT		Colony PCR
AMIS_69690-CF	AGCCAGCTCGAGAGCCTGAA		Colony PCR
AMIS_69690-CR	TGGCATCGGGCTGTTCATCA		Colony PCR
AMIS_68180HBTc-soeF	GGCTGGCGCCTGTTCAAGAAGATCTCGCTCCCGGTCACCGGCGCGCC		Construction of *sspA* -HiBiT
AMIS_68180HBTc-soeR	CTTCTTGAACAGGCGCCAGCCCGACACGGTGCTCCCGGAGCTGACGC		Construction of *sspA* -HiBiT

[a] The recognition sequences for restriction enzymes are underlined.

Table S2. Mutations identified in the suppressor strains[a,b]

Strain	Genomic position	Gene ID[c]	Gene product	Reference[d]	Allele[d]	Mutation type[e]	Amino acid change	Depth[f]	Frequency (%)[g]
S-1	210,266	AMIS_1840	Unknown	T	C	SNV	L71P	115	99.1
	245,680	AMIS_2200	SNF2-family helicase	C	T	SNV	E681K	189	100
	4,469,352	AMIS_42640	LysR-family transcriptional regulator	T	C	SNV	S250G	172	100
	4,936,155	AMIS_47100	ABC transporter ATP-binding protein	G	C	SNV	A174G	134	100
	5,041,117	AMIS_48090	Hypothetical protein of radical SAM superfamily	T	G	SNV	F249C	169	100
	5,643,408	AMIS_53110	Unknown	C	T	SNV	E189K	175	98.3
	6,744,847	AMIS_62710	Histidyl-tRNA synthetase	C	T	SNV	_[h]	131	100
	7,388,624	AMIS_68180	Unknown (SspA)	C	T	SNV	G181S	107	100
	7,388,629			C	T	SNV	G179E	105	100
	8,598,413	AMIS_79540	Two-component system sensor kinase	C	T	SNV	P248S	158	100
	8,598,803			C	T	SNV	P378S	226	98.7
	8,600,797	AMIS_79570	Unknown	T	C	SNV	L287P	195	100
S-2	2,554,361-2,554,362	AMIS_24410	Hypothetical protein containing von Willebrand factor type A domain	AA	GC	MNV	K89A	166	100
	5,008,341	AMIS_47750	5'-3' exonuclease	T	G	SNV	L292R	161	100
	6,432,651	AMIS_60090	Glycosyltransferase	A	G	SNV	K458E	128	100
	7,551,191	AMIS_69690	Unknown	C	T	SNV	Nonsense[i]	157	97.5

[a] Mutations identified in the Δ*asfR* strain, whose genome sequence was analyzed in parallel, were eliminated.

[b] The mutation within *sspA* is shown in orange.

[c] Gene identifiers of the coding sequences where mutations were identified are shown.

[d] Nucleotides on the genomes of the Δ*asfR* and suppressor strains are shown in the "Reference" and "Allele" columns, respectively.

[e] Mutations of single and multiple nucleotide variants are shown as "SNV" and "MNV", respectively.

[f] Counts of the sequence reads on the mutation points are shown.

[g] Proportions of the mutated read counts to total read counts on the mutation points are shown.

[h] A silent mutation of the codon coding for Glu_252 of the gene product was identified.

[i] A nonsense mutation of the codon coding for Gln_88 of the gene product was identified.
